# Book Reviews

**Published:** 1833-05

**Authors:** 


### BIBLIOGRAPHICAL NOTICES.

---

*A Plain Account of Vaccination, designed for the Heads of Families, wherein the History, Advantages, and Errors of this Subject, are popularly treated.* 32mo. pp. 58. Renshaw and Rush, London.

This is a simple, plain, and unpretending little pamphlet, which we doubt not will be of service to those readers for whom it is designed. It will give them in a few words as much information as they require, and quite as much as non-professional people ought to have, upon a subject which cannot fail to be interesting to every parent. It may possibly remove some prejudices, and this will be doing much good. It will probably render intelligible to some well-meaning but ill-judging persons the causes of occasional vaccine failures, and convince them that such failures are not only in the majority of cases, not attributable to their medical attendants, but not even to the ordinary influence of this disease; but to a variety of extraneous and accidental circumstances, which, knowing their power, they will be more easily able to guard against.

---

*Observations on Impediments of Speech, with some Remarks on their successful Treatment; in a Letter addressed to* T. J. Pettigrew, Esq. President to the Westminster Medical Society, &c. By Richard Cull. 8vo. pp. 31. Renshaw and Rush, London.

We do not find any thing strikingly novel, or particularly important, in this pamphlet. It is, however, a short and sensible letter on a very interesting subject: and, as it may probably fall into the hands of some of those unfortunates whose miseries are remediable, if they would but endeavour to cure themselves, it will doubtless convey consolation to them, and will do much good if it encourage them to persevere, *on principle*, in remedying one of the most serious inconveniences which men mixing in this busy world can suffer, viz. the difficulty of communicating their thoughts to others.

Impediments of speech are so often entirely owing to the want of knowledge how to use the vocal organs, that our only surprise is that they have not been made by every stammerer an especial study. So much more frequently does stuttering depend upon an *improper use* of the instruments of speech, than upon any physical defect in the instruments themselves, that we have never known a stammerer who really set about learning the use of the apparatus with half the energy a mechanic strives to master the tools of his trade, that did not get over his impediment of speech. On the contrary, we have known several, whose hesitation was so great as to be absolutely distressing, so completely to gain the mastery over the defect as to become not only intelligible, but fluent, nay even eloquent, speakers. We are therefore quite intolerant of any empirical schemes, or secret systems, by which stammering is pretended to be cured. It must be treated like other defects, either functional or organic, on principle; and those surgeons will be the most likely to afford relief, and treat the disease effectually, who understand the mechanism of the parts, and have, like Mr. Cull, studied their several uses.

